# The forgotten flies: the importance of non-syrphid Diptera as pollinators

**DOI:** 10.1098/rspb.2014.2934

**Published:** 2015-04-22

**Authors:** Katherine A. Orford, Ian P. Vaughan, Jane Memmott

**Affiliations:** 1 School of Biological Sciences, University of Bristol, Bristol Life Sciences Building, 24 Tyndall Avenue, Bristol BS8 1TQ, UK; 2 Cardiff School of Biosciences, Cardiff University, Museum Avenue, Cardiff CF10 3AX, UK

**Keywords:** non-syrphid Diptera, pollinators, pollen-loads, pollen-transport networks, Syrphidae

## Abstract

Bees, hoverflies and butterflies are taxa frequently studied as pollinators in agricultural and conservation contexts. Although there are many records of non-syrphid Diptera visiting flowers, they are generally not regarded as important pollinators. We use data from 30 pollen-transport networks and 71 pollinator-visitation networks to compare the importance of various flower-visiting taxa as pollen-vectors. We specifically compare non-syrphid Diptera and Syrphidae to determine whether neglect of the former in the literature is justified. We found no significant difference in pollen-loads between the syrphid and non-syrphid Diptera. Moreover, there was no significant difference in the level of specialization between the two groups in the pollen-transport networks, though the Syrphidae had significantly greater visitation evenness. Flower visitation data from 33 farms showed that non-syrphid Diptera made up the majority of the flower-visiting Diptera in the agricultural studies (on average 82% abundance and 73% species richness), and we estimate that non-syrphid Diptera carry 84% of total pollen carried by farmland Diptera. As important pollinators, such as bees, have suffered serious declines, it would be prudent to improve our understanding of the role of non-syrphid Diptera as pollinators.

## 1. Introduction

Pollinators play a crucial role in ecosystems by facilitating plant reproduction [1]. They provide an essential ecosystem service, being responsible for 35% of global crop-based food production [2]. Given the recent substantial losses of pollinators [3,4] induced by habitat loss, altered land use, alien species and climate change [5,6], there is a real need for land managers to conserve wild pollinator communities.

Non-syrphid Diptera are diverse, common and ubiquitous in both natural and managed habitats [7,8], and therefore have the potential to contribute significantly to pollination. Although they are unlikely to be the most important pollinators, *en masse* they could have a larger role than previously realized. Seventy-one families of Diptera contain flower-visitors, and Diptera are regular visitors to at least 555 plant species [9], which include over 100 cultivated plant species comprising important crops, such as mango [10], oil seed rape [11], onion [12] and cocoa [13]. Although records of Diptera as flower-visitors exist, evidence of their importance as pollinators is limited.

Unfortunately, studies of pollinator communities usually focus on bumblebees, honeybees, solitary bees (Hymenoptera), hoverflies (syrphid Diptera) and butterflies (Lepidoptera). Consequently, agri-environment schemes and other management strategies are primarily designed to conserve these taxa [14]. Non-syrphid Diptera have received much less attention and are often excluded from key pollination studies [4,6,15–21], probably because they are difficult to identify and assumed to be unimportant. This assumption is untested, however, as there have been no community-wide studies quantifying their contribution to pollination. Some visitation network studies do include non-syrphid Diptera [8,22–24], but not all [25], and those that do rarely measure pollination. Although the neglect of non-syrphid Diptera has been acknowledged [26], there is a paucity of studies that aim to evaluate their relative importance.

One area where the importance of non-syrphid dipteran pollinators is acknowledged is at high altitudes and latitudes, for example in alpine and subarctic ecosystems where bees are less abundant [8,27–29]. Additionally, the sapromyophilous pollination syndrome (sapromyophiles are attracted to flowers mimicking the odours of dead animals or dung) provides good evidence for a significant role of the non-syrphid Diptera in pollination. This pollination syndrome has shaped the flower morphology of a diverse group of angiosperms [9].

In this study, the potential importance of various flower-visitor taxa as pollinators is compared with data originating from a range of temperate ecosystems, including meadows, sand dunes, farmland, heathland and patches of semi-natural vegetation. We estimate their likely importance in farmland habitats in more depth, where the ecosystem service of pollination is required for food production. We specifically compare the syrphid and non-syrphid Diptera to determine whether neglect of the latter is justified.

Visitor identity, visitation, morphology, behaviour, pollen-load, delivery of pollen to stigmas and seed-set are all ways of assessing pollinator importance [9,22,30,31]. In this study, we concentrate on the quantitative side of the pollination process *sensu* Herrera [30], focusing on visitation and pollen-load components. To do this, we use data from existing independent visitation and pollen-transport networks. While pollen transport and visitation do not prove pollination, they are essential prerequisites [32,33].

There are four objectives to our study: (i) to compare pollen-loads (count of grains) of various flower-visiting insect taxa—following findings by Rader *et al.* [34], we predicted the Hymenoptera will have the largest pollen-loads relative to other taxa; (ii) to compare the non-syrphid Diptera and syrphids as pollen-vectors in more detail, considering their specialization in terms of the pollen they transport and their interaction evenness within plant communities; (iii) to compare the abundance and diversity of syrphids and non-syrphid Diptera in agricultural habitats; and (iv) to estimate the relative amount of pollen transported by Syrphidae and non-syrphid dipteran communities in agricultural habitats.

## 2. Material and methods

Our analysis incorporated data from 11 independent projects comprising a total of 71 plant–pollinator-visitation networks and 30 pollen-transport networks (electronic supplementary material, table S1). Together these characterize the interactions between 9082 flower-visitors (520 species) and 261 plant species. The visitation networks quantified which insect species visited which plant species and the pollen-transport networks quantified the number and identity of pollen grains on the insects' bodies. Few studies have collected quantitative pollen-load data at the community level; therefore, this study is limited to the studies cited in electronic supplementary material, table S1. The data were gathered using a standard methodology, this reducing the variation between studies. We concentrated on temperate ecosystems within the UK (with the exception of one Australian study) as dictated by the available data; although the datasets originate from a range of habitats (electronic supplementary material, table S1) most are from farmland.

We collated the network data into four datasets. The first dataset comprised 18 pollen-transport networks from five projects providing pollen-load data at the individual level (3717 pollinators; 404 pollinator species and 61 plant species; Objectives 1 and 2). The second dataset comprised 30 independent pollen-transport networks from eight projects (450 pollinator species and 230 plant species) providing pollen-load data at the pollinator species-level (Objective 2). The third dataset consisted of 71 visitation networks from all 11 studies (Objective 2). The fourth dataset comprised visitation data from 33 independent farms from six agricultural projects (Objectives 3 and 4).

### (a) Objective 1. Pollen-loads of flower-visiting insect taxa

The median count of pollen grains per individual insect was calculated for each species of the Hymenoptera, Coleoptera, Diptera and Lepidoptera for each of the 18 networks. Some orders were subdivided, resulting in nine groups: Hymenoptera were subdivided into pollinator groups; honeybees (*Apis melifera*), bumble-bees (*Bombus* sp.) and solitary bees, and Diptera were divided into the Syrphidae and non-syrphid Diptera. A general linear mixed-effects model (GLMM) with package lme4 [35]) in the R statistical environment fitted with normal errors and identity link was used to determine the difference in pollen-loads (i.e. pollen-grain count; response variable—log_e_ transformed) between the different taxa (fixed factor). Post-hoc Tukey tests with package multcomp [36] were used.

Four additional variables were included in the model (and subsequent models) to account for additional sources of variation: ‘Habitat’, ‘Location’, ‘Sampling’ and ‘Study’. These were incorporated as random factors in the analyses except where the number of levels was less than 5, where fixed effects were used instead [37] (electronic supplementary material, tables S1 and S2 for details of GLMMs). Conditional *R*^2^ (variance explained by both fixed and random factors) and marginal *R*^2^ (variance explained by fixed factors) are reported.

### (b) Objective 2. Pollen specialization and interaction evenness of the dipteran groups

Syrphidae and non-syrphid species' interaction specialization with the lower trophic level (specialization relating to pollen species carried) was assessed using the ‘d’ statistic (package bipartite) [38] within each of the 30 pollen-transport networks. Measures of ‘d’ range from 0 (no specialization) to 1 (perfect specialist). Differences in pollen specialization were determined by a GLMM (normal errors, identity link).

We also compared interaction evenness (Shannon's evenness; a measure of the equitability of visits between visitors and their interacting species [39]) between syrphid (*n* = 1923) and non-syrphid Diptera (*n* = 4776) visitation networks (package bipartite). Interaction evenness equals 1 when the plant–pollinator interactions are uniformly distributed between species. Separate matrices were created for the Syrphidae and non-syrphid Diptera from each visitation network (species-level visitation data) and evenness calculated per network. Differences in interaction evenness between the syrphid and non-syrphid Diptera were determined by a GLMM (normal errors, identity link).

### (c) Objective 3. The abundance and diversity of syrphid and non-syrphid Diptera in farmland

Data from 33 independent farms from six studies were used to compare the abundance (count of insects) and species richness (count of species) per farm (response variables) of the syrphid and non-syrphid Diptera (fixed factor) using GLMMs (Poisson errors). An observation-level random effect was added to both models to create a Poisson-lognormal model accounting for overdispersion [40]. As species richness is likely to increase with the number of individuals captured, we performed a rarefaction analysis to standardize for variable network sizes. Rarefaction allowed the calculation of species richness for a given number of individual samples [41] and was calculated using the vegan package in R. Species richness estimates were compared with a GLMM (normal errors, identity link). GLMMs for Objectives 3 and 4 included ‘farm’ as an additional random factor.

### (d) Objective 4. Pollen transported by the syrphid and non-syrphid dipteran communities in farmland

Pollen-load data were available for three out of the six studies based in agricultural habitats. Therefore to estimate the relative pollen-carrying capacity of the syrphid and non-syrphid dipteran communities, (i) we calculated the median pollen-loads per individual of syrphid (*n* = 583) and non-syrphid Diptera (*n* = 632) from the three farm studies; (ii) we then multiplied these values by the abundance of each dipteran group for each of the 33 farm datasets. Differences between the two groups were investigated using a GLMM (Poisson errors with an observation-level random effect).

## 3. Results

### (a) Objective 1. Pollen-loads of flower-visiting insect taxa

**Figure 1.**
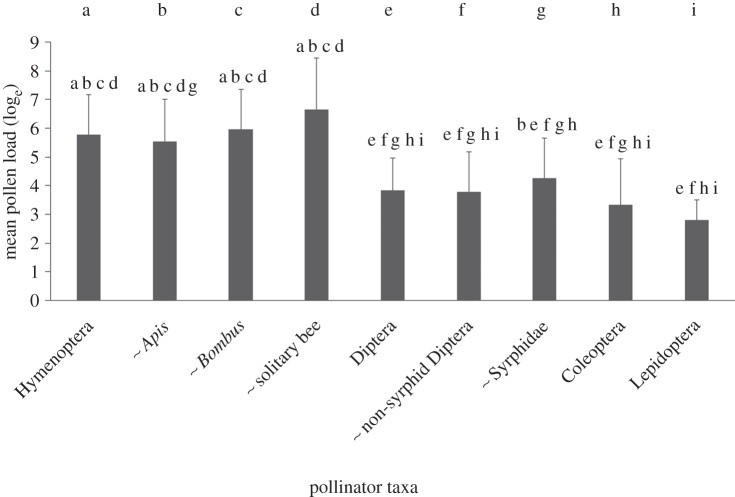
Means (±s.d.) of the log_e_-transformed pollen-load data (count of pollen grains per individual insect) analysed for Objective 1: Hymenoptera (*n* = 2201), separated into *Bombus* (*n* = 901), *Apis* (*n* = 1138) and solitary bees (*n* = 115); Diptera (*n* = 998), separated into the Syrphidae (*n* = 609) and non-syrphid Diptera (*n* = 389); Coleoptera (*n* = 447); and Lepidoptera (*n* = 71) across 18 pollen-transport networks. Pollinator groups with shared letters have no significant difference in pollen-loads.

There was a significant difference in pollen-loads between the flower-visitor taxa (*χ*^2^ = 104.18, d.f. = 8, *p* < 0.001, *R*^2^m = 0.48, *R*^2^c = 0.53 [42]; figure 1; electronic supplementary material, table S2). The Hymenoptera carried the largest pollen-loads; but within this taxon, there was no significant difference between the bumble-bees, solitary bees and honeybees (figure 1). Within the Diptera, there was no significant difference between the Syrphidae and non-syrphid Diptera (figure 1). The pollen-loads of the Syrphidae did not differ significantly from the honeybees; however, the Syrphidae had significantly lower pollen-loads than the other hymenopteran sub-groups. The non-syrphid Diptera had lower pollen-loads than all the hymenopteran sub-groups (figure 1). The Coleoptera and Lepidoptera had significantly lower pollen-loads than all hymenopteran groups, but did not differ significantly from each other (figure 1). These two groups did not differ from the dipteran groups, with the exception of the Lepidoptera having lower pollen-loads than the Syrphidae (figure 1).

### (b) Objective 2. Pollen specialization and interaction evenness of the dipteran groups

The Syrphidae and non-syrphid Diptera did not differ in specialization (0.24 and 0.21, respectively) in the pollen-transport networks (*χ*^2^ = 3.07, d.f. = 1, *p* = 0.080, *R*^2^m = 0.26, *R*^2^c = 0.65; electronic supplementary material, table S2). The Syrphidae had significantly higher interaction evenness (0.65) in the visitation networks than the non-syrphid Diptera (0.61) (*χ*^2^ = 10.65, d.f. = 1, *p* = 0.001, *R*^2^m = 0.38, *R*^2^c = 0.91; electronic supplementary material, table S2).

### (c) Objective 3. The abundance and diversity of syrphid and non-syrphid Diptera in farmland

**Figure 2.**
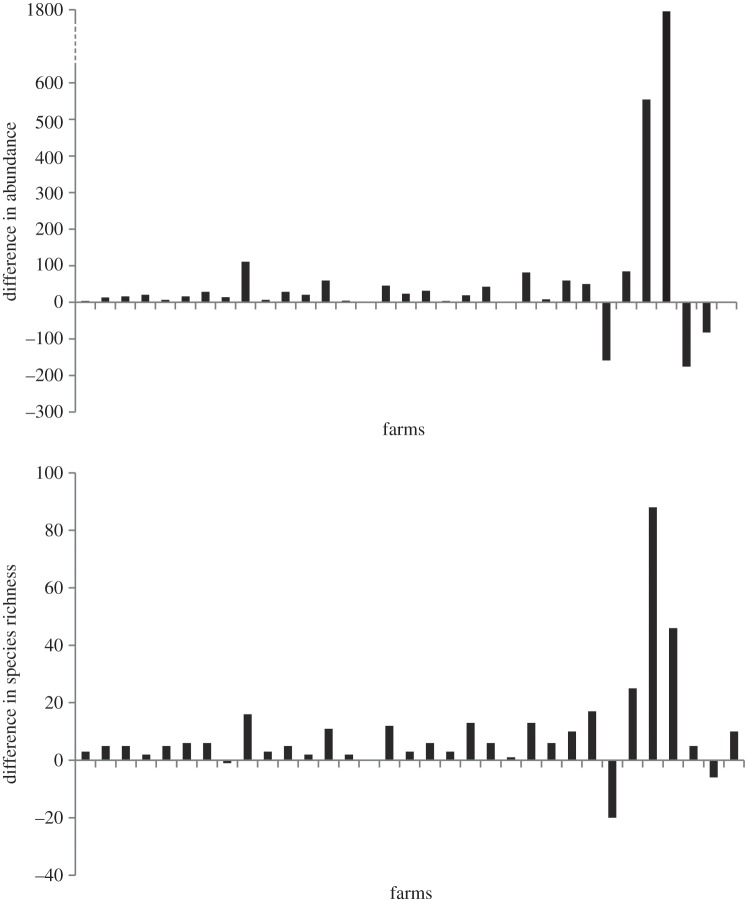
Absolute differences in (*a*) total abundance and (*b*) species richness between the Syrphidae and non-syrphid Diptera found on each of the 33 farms (each bar represents a farm). Positive values show higher abundance or species richness of the non-syrphid Diptera than the Syrphidae.

Non-syrphid Diptera were significantly more abundant than the Syrphidae in agricultural habitats; a median of 28 and six insects were recorded per farm respectively (*χ*^2^ = 24.29, d.f. = 1, *p* < 0.001, *R*^2^m = 0.21, *R*^2^c = 0.83; figure 2; electronic supplementary material, table S2). On average, the non-syrphid Diptera made up 82% (*s* = 23%) of the dipteran abundance recorded on the farms. Species richness of non-syrphid Diptera was also higher than the Syrphidae; a median of seven and three species per farm, respectively (*χ*^2^ = 27.08, d.f. = 1, *p* < 0.001, *R*^2^m = 0.15, *R*^2^c = 0.88; figure 2; electronic supplementary material, table S2). On average non-syrphid Diptera made up 73% (*s* = 19%) of dipteran species. Following rarefaction, the species richness of the non-syrphid Diptera was still greater than the Syrphidae (*χ*^2^ = 23.27, d.f. = 1, *p* < 0.001, *R*^2^m = 0.055, *R*^2^c = 0.94); therefore, patterns detected were unlikely to be driven by sampling effects. Together the dipteran groups made up 67% of the total abundance and 66% of the total species richness of all flower-visitors in the farm networks.

### (d) Objective 4. Pollen transported by the syrphid and non-syrphid dipteran communities in farmland

Median pollen-load for the Syrphidae and non-syrphid Diptera in the agricultural habitats was seven and 16 pollen grains, respectively; this was multiplied by dipteran abundance counted in each of the farms. The non-syrphid Diptera communities carried significantly more pollen than the Syrphidae (*χ*^2^ = 43.79, d.f. = 1, *p* < 0.001, *R*^2^m = 0.33, *R*^2^c = 0.80; electronic supplementary material, table S2); 84% of all dipteran-carried pollen was carried by the non-syrphid Diptera.

## 4. Discussion

To our knowledge, this is the first study to highlight the potential importance of non-syrphid Diptera as pollinators using a network approach at a multi-family, multi-habitat level. The syrphid and non-syrphid Diptera did not significantly differ in their pollen-loads. There was no difference in pollen-transport specialization between the two groups. However, the Syrphidae had significantly greater visitation evenness in the visitation networks. The non-syrphid Diptera made up the majority of the flower-visiting Diptera in agricultural habitats, and we estimate that they carry 84% of total pollen carried by farmland Diptera.

Our study is limited to temperate ecosystems (predominantly UK farmland) due to the availability of data, and consequently the results should be considered in this context only. If tropical systems were included it is possible that different conclusions would be drawn as multi-latitudinal studies on plant–pollinator networks have revealed differences in network structure between temperate and tropical climates (e.g. specialization) [43].

### (a) The pollen-loads of the different flower-visiting taxa

As predicted, the Hymenoptera carried the highest pollen-loads. Bees make many visits to flowers to provision their broods, and many have specialized structures for pollen transport [26]. Although bees are acknowledged to be highly effective pollinators [44], many species are in decline. Most widely reported are honeybee populations—primarily a result of heavy pathogen and parasite loads, pesticide use and diminishing resources [5,6,45]. Declines have also been observed for many wild pollinator species, though this rate of decline has slowed or reversed for several species [3,5,6]. Ecological conditions and anthropogenic pressures affecting bees may differ from those affecting flies due to the differences in their ecology [46], and it is possible that these alternative pollinator taxa could provide some insurance against bee losses. Many families of Diptera, including the Muscidae and Scathophagidae, have bristles that trap pollen; the Bombyliidae are furry and the Acroceridae are thought to have hairs adapted for carrying pollen [7]. Indeed, the average pollen-load of the Diptera was second to that of the Hymenoptera, this being in agreement with the findings of Rader *et al.* [34]. In this study, the Syrphidae pollen-loads did not significantly differ from *Apis,* this strongly suggesting that Dipteran groups could be important as pollinators.

The ‘insurance value’ of Diptera is conditional on the fly populations having similar functional attributes (e.g. mouth parts, feeding behaviour and phenology) to fill the niche of declining bee species. Bombyliidae flies have long tongues, which can pollinate flowers possessing long-tube corollas; however, the presence of this group in our dataset was low (just 13 individuals). Ideally, functional diversity analyses should be performed in order to determine whether Diptera could compensate for bee declines. Unfortunately, though, trait data for many dipteran species is currently lacking, in part because their importance as pollinators is often overlooked.

### (b) The syrphid and non-syrphid Diptera as potential pollinators

Pollen-loads (number of grains) did not differ significantly between the syrphid and non-syrphid Diptera. As an insect's pollen-load influences the likelihood of pollen being transferred to stigmas [32,33], the syrphids and non-syrphids may not differ in their efficacy as pollinators. Thus, it may be premature to dismiss the non-syrphid Diptera in pollination studies on the grounds that, unlike the Syrphidae, they are unimportant. That said, further research, especially to measure seed-set following visits by specific taxa, is required to confirm this. Indeed, a limitation of our approach is our focus on the visitation and pollen-transport stages of the pollination process. The most comprehensive way of assessing pollinator importance would be to assess their relative influences on seed-set. This would require bagging of replicate flowers after single visits by each flower-visiting species—a challenging approach at the community level.

There was no difference in specialization of the non-syrphid Diptera and the Syrphidae in terms of the identity of pollen transported. Pollen specialization has implications for the pollination of plant communities. More generalized pollen-transfer gives the potential to pollinate a greater diversity of species, although pollination may be less effective [47]. The Syrphidae had greater interaction evenness and this has potential implications for the overall stability of the plant–pollinator community; higher interaction evenness is associated with stability [48].

### (c) Non-syrphid dipteran abundance and diversity in agro-ecosystems

The greater richness of the non-syrphid Diptera found in agro-ecosystems could provide a more stable pollination service as richness has been positively associated with the stability of ecosystem processes [49,50]. We estimated that the non-syrphid Diptera carried 84% of the dipteran pollen in farmland habitats. Considering Diptera made up 67% of all flower-visitor abundance in the farm networks, this is a significant proportion of the pollen transported in farmland. Unlike many bee species, the non-syrphid Diptera have not been widely reported to be threatened by current agricultural practices, although it is possible that any declines have been overlooked, and further studies are needed to assess their vulnerability.

## 5. Conclusion

Our analysis of pollen-transport and visitation networks strongly suggests that it is inappropriate to exclude non-syrphid Diptera from pollination studies. Looking forward, our assessment of pollinator importance *sensu* Herrera [30] needs to be augmented in the future with pollen-transfer and ultimately seed-set analyses using controlled experiments. Per-visit effectiveness of non-syrphid dipteran species for crops and wild plants should be assessed focusing on families that may fill the niche of declining bees such as the Bombyliidae. More generally, training in dipteran taxonomy should be more available to ecologists. Alternatively, specialist taxonomists should be included in research projects to prevent pollination biologists being deterred from recording Diptera due to identification difficulties. Given the current declines in Hymenoptera, along with large unknowns such as the effect of climate change on pollinators, improving our understanding of the role of the less well-known pollinator groups is timely.

## Supplementary Material

Table S1 and table 2

## Data accessibility

The data used in this paper can be accessed via Dryad: doi:10.5061/dryad.41m4d.
