# Supplementary material for: The forgotten flies: the importance of non-syrphid Diptera as pollinators
Source: Proc Biol Sci. 2015 Apr 22;282(1805):20142934. doi: 10.1098/rspb.2014.2934 (PMC4389612; doi:10.1098/rspb.2014.2934)
Supplement: Table S1 and table 2 [file rspb20142934supp1.docx]

**Table S1.** The details of the original studies including study ID, habitat, transect/plot size, number of surveys, months sampling, geographic area, number of visitation (V) and pollen transport (P) networks and finally whether data are given at individual or species level. Few studies have collected quantitative pollen-load data at the community level, therefore this study is limited to the studies cited in Table 1. As these datasets are from the Memmott group they are gathered using a standard methodology reducing variation between studies. In the original studies the visitation networks quantified which insect species visited which plant species from field observations; the pollen-transport networks quantified the number and identity of pollen grains found on the insects in these networks. To make the pollen transport networks insects were systematically dabbed with fuchsin pink gel; pollen storage areas were avoided because these contain pollen unlikely to be available for pollination. *agricultural studies included in Objectives 3 and 4.

| **Study** | **Habitat** | **Sampling:Transect/**  **Plot size** | **Number of surveys** | **Months sampling** | **Geographic area** | **# visitation (V) and pollen transport (P) networks** | **Data at individual or species level?** |  |
| --- | --- | --- | --- | --- | --- | --- | --- | --- |
| Chateil C. & Memmott, J. (2005) (unpublished data)* | Farmland | Transects 30m x 2m | 2 per farm (40 total) | Apr-Jun 2005 | South West England | 20 (V)  0 (P) | Species | |
| Downe C. & Memmott J. (1999) (unpublished data) | Sand dune | Plots  100m x 170m | 16 | April-Aug 1999 | Wales | 1 (V)  1 (P) | Individual | |
| Forup, M., Henson, K., Craze, P. & Memmott, J. (2008) *Journal of Applied Ecology***,** 742-752 | Heathland | Transects 100m x 2m | 8 (2001), 8 (2004) | Apr-Sep 2001,  May-Sep 2004 | South West England | 8 (V)  8 (P) | Individual | |
| Forup, M. & Memmott, J. (2005) *Restoration Ecology***,** 265-274 | Meadows | Transects 50m x 2m | 6 (3 meadows), 4 (1 meadow) | May-Jun/July (depending on cutting date of meadow) 2000 | South West England | 3 (V)  3 (P) | Individual | |
| Gibson, R., Nelson, I., Hopkins, G., Hamlett, B. & Memmott, J. (2006). *Journal of Applied Ecology***,** 246-257* | Arable | Plots  1200-1250 m^2^ | 172, 192, 176, 240, 120 sampling events for the five sites. | Jun-Sep 2002 | South West England | 5 (V)  5 (P) | Individual | |
| Gibson & Memmott unpublished data (2009)* | Arable | Transects 25m x 2m | 24 | Mar-Sep 2009 | South West England | 3 (V)  3 (P) | Species | |
| Lopezaraiza-Mikel, M., Hayes, R., Whalley, M. & Memmott, J. (2007) *Ecology Letters***,** 539-550. | Patches of natural or semi-natural vegetation in an urban environment | Transects 65m x 2m – 90m x 2m | 8 | Jul-Sep 2003 | South West England | 8 (V)  8 (P) | Species | |
| Pocock, M., Evans, D. & Memmott, J. (2012) *Science,* **335,** 973-977* | Farmland | Transects  25m x 1m (2007)  50m x 1m (2008) | 3-4 surveys in each of the 11 habitats per month (a total of 361 transects) | Mar-Sep 2007  May-Aug 2008. | South West England | 1 (V)  1 (P) | Individual | |
| Orford, Murray, Vaughan & Memmott, (2011) unpublished data* | Conventional grassland | Plots 500m^2^ | 576 surveys | May-Sep 2011 | South West England | 1 (V)  0 (P) | Individual | |
| Orford, Murray, Vaughan & Memmott, (2012) unpublished data* | Conventional grassland | Plots 500m^2^ | 5 surveys per pasture (100 in total) | Apr-Aug 2012 | South West England | 20 (V)  0 (P) | Individual | |
| Nelson & Memmott, (2006) unpublished data | Coastal heath | Transects 100m x 2m | 4 surveys per transect | Sep-Dec 2005 | Australia | 1 (V)  1 (P) | Species | |

Table S2. Outputs of the GLMM models for each Objective, including the estimated coefficients for both random and fixed factors in the models. For random factors the group-level standard deviation (s.d.) is shown along with the estimated factor levels, whilst for fixed factors the estimated coefficients are provided with the standard error in parentheses. ‘Taxon’ was the main focus of the analysis and is fully described in the main text. ‘Study’ and ‘Habitat’ were included as random factors except where the number of habitat types was less than five (Objectives 3 and 4), in which case a fixed effect was used instead [1]. ‘Farm’ (33 levels) was included as a random factor in Objectives 3 and 4, but only the group-level standard deviation is given as the values for individual farms are uninformative. 'Sampling' was included as a fixed factor in all models: the coefficient estimates are for ‘Transect’ relative to ‘Plot’ design. The fixed factor ‘Location’ had three levels (‘Australia’, ‘SW England’ and ‘Wales’) and was not included in the models for Objectives 3 and 4 as all studies were based in SW England. The coefficients for 'Location' are ‘SW England’ and ‘Wales’ relative to ‘Australia’ (when 3 levels) or ‘Wales’ relative to ‘SW England’ (when 2 levels). When ‘Habitat’ was included as a fixed factor (Objectives 3 and 4), the coefficients are for ‘Conventional grassland’ and ‘Farmland’ relative to ‘Arable’. For the fixed factor ‘Taxon’ the coefficients are for ‘Syrphidae’ relative to ‘Non-syrphid Diptera’ in Objectives 2-4 whilst for Objective 1 the coefficients for the different taxonomic groupings are relative to ‘Apis’.

| Objective 1- Pollen loads | |
| --- | --- |
| Random factors | |
| **Study** (s.d. = 0.028) | **Habitat** (s.d. = 0.41) |
| Downe 1999= <0.001  Forup 2008= <-0.001  Forup 2005= 0.046  Gibson 2006= <-0.001  Pocock 2012= 0.014 | Arable= <0.001  Farm= 0.082  Heathland= -0.34  Meadow= 0.27  Sand dune= <-0.001 |
| Fixed factors | |
| **Taxon**  Bombus = 0.42(0.45)  Coleoptera = -2.22(0.45)  Diptera = 1.71(0.45)  Hymenoptera = 0.24(0.45)  Lepidoptera = 2.72(0.45)  Non-syrphid Diptera = -1.75(0.45)  Solitary bee = 1.12(0.45)  Syrphidae 1.29(0.45) | **Sampling**  Transect = 0.93(0.59)  **Location**  Wales = -0.08(0.80) |

| Objective 2- Pollen Specialisation (‘d’) | |
| --- | --- |
| Random factors | |
| **Study** (s.d. = 0.081) | **Habitat** (s.d. = 0.085) |
| Downe 1999= <0.001  Forup 2008= -0.032  Forup 2005= 0.039  Gibson 2006= <0.001  Gibson 2009= -0.0091  Lopezaraiza-Mikel 2007= -0.054  Nelson 2006= <0.001  Pocock 2012= 0.056 | Arable= 0.009  Coastal Heath= <0.001  Farm= 0.062  Heathland= -0.035  Hay meadow= 0.043  Sand dune= <0.001  Semi-natural in urban= -0.060 |
| Fixed factors | |
| **Taxon**  Syrphidae = 0.096(0.055) | **Sampling**  Transect = -0.22(0.14)  **Location**  SW England = 0.06(0.15)  Wales = 0.085(0.20) |

| Objective 2- Evenness | |
| --- | --- |
| Random factors | |
| **Study** (s.d. = 0.01) | **Habitat** (s.d. = 0.058) |
| Chateil 2005= <0.001  Downe 1999= -1.47  Forup 2008= -0.011  Forup 2005= 0.007  Gibson 2009= 0.004  Gibson 2006= <0.001  Lopezaraiza-Mikel 2007= -0.051  Nelson 2006= 1.55  Orford 2011= 0.024  Orford 2012= 0.029  Pocock 2012= -0.001 | Arable= 0.023  Coastal Heath= <0.001  Conventional Grassland= -0.076  Farm= -0.043  Heathland= 0.029  Meadow= -0.013  Sand dune= <0.001  Semi natural in urban= 0.079 |
| Fixed factors | |
| **Taxon**  Syrphidae = 0.039(0.01) | **Sampling**  Transect = 0.032(0.023)  **Location**  SW England = -0.13(0.066)  Wales = -0.17(0.086) |

| Objective 3- Abundance | |
| --- | --- |
| Random factors | |
| **Study** (s.d. = 1.61) | **Farm** (s.d. = 0.29) |
| Chateil 2005= -1.88  Gibson 2009= 0.004  Gibson 2006= 0.006  Orford 2012= -1.81  Orford 2011= 1.83  Pocock 2012= 1.97 |  |
| Fixed factors | |
| **Taxon**  Syrphidae = -1.25(0.23) | **Sampling**  Transect = 0.39(2.35)  **Habitat**  Conventional Grassland = 0.46(2.04)  Farmland = -1.47(2.04) |

| Objective 3- Species richness | |
| --- | --- |
| Random factors | |
| **Study** (s.d. = 1.07) | **Farm** (s.d. = 0.31) |
| Chateil 2005= -1.47  Gibson 2009= 0.014  Gibson 2006= 0.022  Orford 2012= -0.87  Orford 2011= 0.92  Pocock 2012= 1.55 |  |
| Fixed factors | |
| **Taxon**  Syrphidae = -0.81(0.14) | **Sampling**  Transect = 0.55(1.55)  **Habitat**  Conventional Grassland = 0.071(1.35)  Farmland = -0.91(1.35) |

| Objective 3- Rarefaction | |
| --- | --- |
| Random factors | |
| **Study** (s.d. = 7.46) | **Farm** (s.d. = 1.43) |
| Chateil 2005= -7.04  Gibson 2009= <-0.001  Gibson 2006= <0.001  Orford 2012=-1.99  Orford 2011= 1.99  Pocock 2012= 7.04 |  |
| Fixed factors | |
| **Taxon**  Syrphidae = -2.73(0.48) | **Sampling**  Transect = 3.46(10.67)  **Habitat**  Conventional Grassland = -0.10(9.26)  Farmland = -2.56(9.26) |

| Objective 4- Pollen carried by communities | |
| --- | --- |
| Random factors | |
| **Study** (s.d. = 1.62) | **Farm** (s.d. = 0.46) |
| Chateil 2005= -1.93  Gibson 2009= 0.11  Gibson 2006= -0.11  Orford 2012= -1.73  Orford 2011= 1.75  Pocock 2012= 1.97 |  |
| Fixed factors | |
| **Taxon**  Syrphidae = -2.36(0.28) | **Sampling**  Transect = 0.15(0.96)  **Habitat**  Conventional Grassland = 0.14(1.75)  Farmland = -1.54(1.75) |

Reference

1. Gelman A., Hill J. 2007 *Data Analysis Using Regression and Mulitlevel/Hierarchical Models*. Cambridge University Press, Cambridge.
